# Machine Learning for Intelligent and Adaptive Communication Systems: From Optimization to Emerging Paradigms

**DOI:** 10.3390/s26092882

**Published:** 2026-05-05

**Authors:** Haeyoung Lee, Yichuang Sun, Oluyomi Simpson

**Affiliations:** School of Physics, Engineering and Computer Science, University of Hertfordshire, Hatfield AL10 9AB, UK; y.sun@herts.ac.uk (Y.S.); o.simpson@herts.ac.uk (O.S.)

## 1. Introduction

Machine learning (ML) has been increasingly considered for various communication applications, demonstrating promising feasibility and effectiveness in enhancing system intelligence, adaptability, and operational efficiency [1]. By enabling data-driven modeling and decision-making, ML has been successfully applied to a wide range of communication tasks, including signal processing, resource allocation, and network management [2,3]. These developments have not only improved overall system performance but also strengthened the role of ML as a key enabler in the evolution of future communication systems, moving beyond conventional optimization toward fully autonomous and cognitive network operation [4].

At the same time, communication environments are undergoing rapid transformation, driven by emerging applications and increasingly complex system requirements. Communication systems are expanding across diverse domains, including satellite networks, unmanned aerial vehicle (UAV) systems, vehicular communications, and large-scale Internet of Things (IoT) deployments [5,6,7]. These environments are inherently dynamic, heterogeneous, and data-intensive, making traditional model-based approaches increasingly insufficient. As a result, there is a growing need for intelligent mechanisms capable of adapting to rapidly changing conditions [1]. In this context, ML is expected to take on a more active role in enabling the real-time, adaptive, and context-aware optimization of communication systems.

Despite its strong potential, the application of ML in communication systems also introduces several critical challenges [8,9]. These include the limited availability of high-quality data, concerns related to data privacy and security, and the need for efficient learning across distributed and resource-constrained environments [10,11]. Addressing these challenges has led to the emergence of new learning paradigms, such as federated learning and edge learning, which enable distributed and privacy-preserving model training. In addition, large-scale AI models and privacy-preserving techniques are being explored to enhance learning capability while maintaining scalability and data confidentiality, further supporting the deployment of ML in complex communication scenarios.

Beyond algorithmic advancements, ML is also driving the development of novel communication and computing architectures. In particular, emerging hardware-oriented solutions, such as optical neural networks and optical convolution accelerators, are being investigated to overcome the limitations of conventional electronic processing [12,13]. These architectures leverage the properties of light to perform computation, offering significant improvements in processing speed and energy efficiency. Such innovations highlight the growing convergence of communication, computation, and intelligence, and point toward new system designs that are inherently optimized for ML-driven operations.

Given these developments, the interest in integrating ML into communication systems is expected to continue growing. In this context, and following the progress established in the first edition of this Topic [14], this second edition further extends the exploration of ML toward more complex, large-scale, and heterogeneous communication environments. Compared to the first edition, which demonstrated the feasibility and broad applicability of ML across core communication tasks, this edition places greater emphasis on advanced learning paradigms, scalability, and integration into next-generation communication and computing architectures, reflecting a natural progression from foundational studies toward more practical and intelligent system designs.

Accordingly, this Editorial presents fourteen contributions that collectively explore the role of ML across a diverse range of applications. The featured works address key aspects including intelligent network optimization, data-driven sensing and monitoring, advanced learning mechanisms, and emerging communication and computing architectures. Together, these contributions provide valuable insights into research directions and highlight the transformative impact of ML on next-generation communication systems.

## 2. An Overview of Published Articles

ML has emerged as a powerful enabler for transforming conventional communication systems into adaptive and intelligent infrastructures. In this context, the following contributions demonstrate how ML can be leveraged to address a broad range of communication challenges, such as signal prediction, dynamic resource allocation, interference mitigation, encompassing anti-jamming strategies and path planning. Apavatjrut (contribution 2) developed a Received Signal Strength Indicator (RSSI) prediction framework by leveraging adaptive supervised learning models that incorporate environmental sensing data to capture non-linear signal variations. The effectiveness of the proposed approach is validated in a LoRa (Long-Range) network environment using real-world sensor measurements. Wang et al. (contribution 3) address anti-jamming communication by integrating generative adversarial networks (GANs) with deep Q-networks (DQNs), enabling data augmentation and improved reinforcement learning convergence. Mawlood et al. (contribution 5) propose a dynamic bandwidth allocation scheme using deep reinforcement learning, specifically a Soft Actor–Critic (SAC) model enhanced with continual learning to adapt to changing traffic conditions, with the aim of improving fairness and overall efficiency in network resource allocation. Suarez del Valle et al. (contribution 6) employ a contextual multi-armed bandit approach to optimize beam selection in mmWave vehicular networks, enabling online learning under dynamic environments. AlMania et al. (contribution 11) designed an energy-efficient UAV path planning framework by combining reinforcement learning with particle swarm optimization (PSO), allowing adaptive navigation in complex environments. Zheng et al. (contribution 13) enhanced modulation recognition in visible light communication (VLC) systems using a deep learning architecture that integrates Convolutional Neural Networks (CNNs), attention mechanisms, and Long Short-Term Memory (LSTM) networks. This integrated design aims to jointly capture spatial features, temporal dependencies, and salient signal characteristics, thereby improving robustness and recognition accuracy under noise, distortion, and dynamic channel conditions. Finally, da Silva et al. (contribution 14) provide a survey of ML-based approaches for PAPR (Peak to Average Power Ratio) reduction in OFDM (Orthogonal Frequency Division Multiplexing) systems.

Building upon these optimization-focused studies, machine learning is further applied to enable intelligent monitoring, detection, and sensing capabilities across communication-related systems. In this direction, four contributions illustrate how data-driven approaches can replace traditional, labor-intensive, or less accurate techniques with more efficient and scalable solutions. Szcerba et al. (contribution 1) applied supervised learning to detect faults in hybrid fiber–coaxial networks, improving service reliability through proactive maintenance. López-Muñoz et al. (contribution 9) present a hybrid system combining RF signal analysis and computer vision for accurate UAV (unmanned aerial vehicle) detection, localization, and tracking, effectively improving detection accuracy and reducing false positives in drone monitoring for security applications. In the context of structural health monitoring, Shah Mansouri et al. (contribution 10) utilized machine learning and statistical techniques to detect and localize cracks in materials using sensor networks. Meanwhile, Ma et al. (contribution 12) address automatic modulation recognition, a critical sensing task in wireless communications, by employing meta-learning to overcome data scarcity challenges. Together, these studies illustrate the growing importance of machine learning in enabling intelligent perception and diagnostics within modern communication ecosystems.

Beyond these application-driven studies, recent efforts have explored advanced machine learning paradigms to address fundamental limitations in communication systems, particularly in terms of data availability and distributed operation. Tang et al. (contribution 4) introduce an over-the-air federated learning framework that enables efficient and privacy-preserving distributed model training while mitigating interference in multi-cell environments. Complementing this, Ma et al. (contribution 12) propose a meta-learning-based approach for automatic modulation recognition, allowing the model to learn how to learn and rapidly adapt to new modulation types with limited training data. These contributions demonstrate how emerging learning paradigms such as federated learning and meta-learning provide scalable, distributed, and data-efficient solutions, paving the way for more intelligent and collaborative communication systems.

Looking ahead, machine learning is also driving the development of novel communication and computing architectures, pointing toward the future of intelligent systems. The study by Tang et al. (contribution 4) represents a shift toward integrated communication learning frameworks through over-the-air federated learning, while AlMania et al. (contribution 11) extend this vision to distributed and autonomous Internet of Drones systems. At the hardware level, Chen et al. (contribution 7) and Xia et al. (contribution 8) introduce optical neural networks and optical convolution accelerators, respectively, offering new computing architectures that significantly improve speed and energy efficiency compared to traditional electronic processing. Chen et al. performed inference directly using light in passive optical systems, while Xia et al. leveraged frequency-domain encoding to enable highly parallel and efficient convolutional operations. Together, these works highlight the potential of optical computing to overcome the performance and energy limitations of conventional ML hardware. Collectively, these advances demonstrate how machine learning is not only enhancing existing systems but also enabling fundamentally new architectures that redefine the boundaries of communication and computation.

## 3. Conclusions

In summary, the contributions presented in this collection demonstrate the transformative impact of machine learning across multiple layers of communication systems, from optimization and control to sensing, learning, and architectural design. The reviewed works highlight how ML enables more adaptive, efficient, and intelligent solutions to longstanding challenges in wireless communications, network management, and system monitoring. At the same time, emerging paradigms such as federated learning and optical computing signal a shift toward more distributed, scalable, and energy-efficient architectures. Despite these advances, several open challenges remain, including model generalization, real-time deployment constraints, and the integration of heterogeneous systems. Future research is expected to further explore the synergy between machine learning and communication technologies, ultimately paving the way for fully autonomous, intelligent, and resilient communication ecosystems.
